# Repeatability of Swan600 and Its Agreement with Lenstar LS900 for Ocular Biometry in Children

**DOI:** 10.3390/diagnostics16203421

**Published:** 2026-10-21

**Authors:** Yuanbin Li, Yunyun Sun, Weibin Chen, Jing Fu

**Affiliations:** Beijing Ophthalmology & Visual Sciences Key Laboratory, Beijing Tongren Eye Center, Beijing Tongren Hospital, Capital Medical University, Beijing 100730, China; yuanbinli2001@126.com (Y.L.); 2008sunshinesyy@163.com (Y.S.); cwbtrhos@126.com (W.C.)

**Keywords:** optical biometry, axial length, pediatric ophthalmology, repeatability, agreement, Bland–Altman analysis

## Abstract

**Background/Objectives**: To evaluate the repeatability of the Swan SW600 optical biometer and its agreement with the Lenstar LS900 in measuring ocular biometric parameters in children and adolescents. **Methods**: This prospective study included 778 children and adolescents aged 3–18 years. Parameters included axial length (AL), corneal radii in the flattest and steepest meridians (R1 and R2), central corneal thickness (CCT), anterior chamber depth (AD), lens thickness (LT), white-to-white distance (WTW), and axial length-to-corneal radius ratio (AL/CR). Repeatability of Swan SW600 was assessed using within-subject standard deviation (Sw), coefficient of repeatability (CoR), coefficient of variation (CoV), and intraclass correlation coefficient (ICC). Agreement between devices was evaluated using paired *t* tests, ICC, and Bland–Altman analysis. **Results**: Swan SW600 repeatability was excellent for AL, R1, R2, CCT, AD, and AL/CR (ICC = 0.9918–0.9999) and lower for LT (ICC = 0.9060) and WTW (ICC = 0.8452). Agreement with Lenstar LS900 was strongest for AL (mean difference −0.050 ± 0.041 mm; 95% LoA −0.131 to 0.031 mm; ICC = 1.000), R1 (ICC = 0.986), R2 (ICC = 0.980), and AL/CR (ICC = 0.993). CCT and AD showed good agreement (ICC = 0.877 and 0.895), whereas LT showed lower agreement (ICC = 0.689). WTW showed poor agreement (ICC = 0.288). Age-stratified analyses showed consistently high agreement for AL, R1, R2, and AL/CR across all age groups, including children aged 3–6 years, whereas agreement for CCT, AD, LT, and WTW was more variable. **Conclusions**: The Swan SW600 demonstrates high repeatability and close agreement with the Lenstar LS900 for AL, corneal radii, and AL/CR in children and adolescents, including preschool-aged children; however, the weaker agreement observed for LT and particularly WTW suggests that these parameters should be interpreted with caution.

## 1. Introduction

Myopia has become a major public health concern, particularly among children and adolescents in East and Southeast Asia [1]. The prevalence of myopia has increased markedly over recent decades, and nearly half of the global population is projected to be myopic by 2050 [2]. In China, the burden is particularly high, with myopia affecting more than half of children and adolescents and more than 80% of high school students [3,4]. Earlier onset and greater progression during childhood increase the lifetime risk of high myopia and associated ocular complications [5,6]. Early identification and accurate monitoring of ocular growth are therefore important for myopia prevention and management in pediatric populations.

Large-scale myopia screening among children, particularly in China, mostly depends on visual acuity assessment and non-cycloplegic refraction [7,8]. However, non-cycloplegic measurements are susceptible to accommodative interference, particularly in younger children, which may result in inaccurate estimation of refractive status [9,10]. Ocular biometric parameters provide complementary and relatively objective information on ocular development. Among these parameters, axial length (AL) is widely used to monitor ocular growth and myopia progression because it provides an objective, non-invasive, and highly repeatable measure of axial elongation [11,12]. The axial length-to-corneal radius ratio (AL/CR) is also closely associated with refractive status and may provide additional information for identifying children at risk of myopia [13,14]. Accurate measurement of AL and other biometric parameters is therefore increasingly important in pediatric ophthalmic assessment and longitudinal myopia monitoring.

The Lenstar LS900 (Haag-Streit AG, Köniz, Switzerland), based on optical low-coherence reflectometry (OLCR), is an established optical biometer with high repeatability for multiple ocular biometric parameters [15,16,17,18]. However, biometric measurements can be challenging in preschool and younger school-aged children because successful acquisition requires stable fixation and adequate cooperation. A device that enables rapid acquisition while maintaining reliable measurements may therefore be particularly useful in pediatric clinical practice and large-scale screening [19]. The Swan SW600 (Anshikang, Changsha, China) is a newly developed optical biometer designed for rapid ocular biometric measurement. According to the manufacturer, it can acquire ocular biometric parameters in approximately one second per eye and incorporates an audiovisual fixation system intended to facilitate cooperation in children.

Given these potential advantages, the measurement performance of the Swan SW600 needs to be compared with that of an established optical biometer, particularly in younger children. Therefore, this study aimed to assess the repeatability of the Swan SW600 and its agreement with the Lenstar LS900 for ocular biometric measurements in children and adolescents, with additional age-stratified analyses to evaluate its performance in preschool-aged children.

## 2. Materials and Methods

### 2.1. Study Design

This prospective, nonrandomized study enrolled 778 children and adolescents with healthy eyes at Beijing Tongren Hospital, Capital Medical University, between September 2024 and June 2025. The study was approved by the Ethics Committee of Beijing Tongren Hospital, Capital Medical University (approval no. TREC2024-KY071; approval date: 29 May 2024), and adhered to the tenets of the Declaration of Helsinki. Written informed consent was obtained from the parents or legal guardians of all participants, and verbal assent was obtained from the children.

The inclusion criteria were the ability to cooperate with ophthalmic examinations, willingness to participate in the study, no history of ocular surgery, and no use of orthokeratology lenses or rigid gas-permeable contact lenses within the previous three months.

The exclusion criteria included the presence of ocular disease, strabismus, previous ocular surgery, or ocular trauma. Before enrollment, all eyes underwent a complete ophthalmologic examination consisting of subjective refraction, noncontact tonometry, and slit lamp evaluation.

### 2.2. Optical Biometers

The Swan SW600 (version 1.0.2; Anshikang, Changsha, China) is an optical biometer based on OLCR. The device uses a Michelson interferometer configuration with a superluminescent diode (SLD) light source with a central wavelength of approximately 850 nm and a broadband spectral width of approximately 100 nm. Ocular biometric parameters are obtained from interference signals reflected at multiple ocular interfaces, including the anterior and posterior corneal surfaces, anterior and posterior lens surfaces, and the retina. These signals enable measurement of AL, central corneal thickness (CCT), anterior chamber depth (AD), and lens thickness (LT). The device incorporates fiber-optic signal transmission and high-speed scanning to facilitate rapid acquisition and stable biometric measurements. Its measurement range extends up to 40 mm in axial length. To improve fixation and cooperation, particularly in pediatric patients, the Swan SW600 is equipped with an audiovisual fixation system. Detailed information regarding the proprietary algorithms and refractive indices used by the device is not publicly available.

The Lenstar LS900 (version 9.6.0; Haag-Streit AG, Koniz, Switzerland) is an established OLCR-based optical biometer. The device uses an 820-nm superluminescent diode laser to measure multiple ocular biometric parameters, including CCT, AD, LT, and AL. Keratometry measurements are obtained by analyzing anterior corneal curvature at 32 reference points distributed over two concentric rings with diameters of approximately 2.30 mm and 1.65 mm. White-to-white distance (WTW) measurements are derived from high-resolution color photography of the anterior segment. The measurement principles and repeatability of the Lenstar have been described previously [18,20,21].

### 2.3. Measurement Protocol

Measurements were obtained with the two devices in randomized order on the same day in a darkened examination room. Although biometric measurements were obtained from both eyes, only the left eye was included in the primary analysis to ensure that each participant contributed a single statistically independent observation and to avoid the statistical dependence associated with inter-eye correlation. The left eye was selected consistently for all participants and was not chosen on the basis of measurement quality or biometric characteristics. Participants were instructed to blink normally before measurement and to open their eyes sufficiently to minimize interference from the eyelids and eyelashes.

One experienced operator performed the examinations according to a standardized protocol. Attempts were made to obtain three consecutive valid measurements with each device. A measurement was considered valid when the instrument generated a numerical biometric value and the acquisition was accepted by the device’s built-in quality-control procedure. No prespecified maximum number of acquisition attempts was set. Repeated acquisition attempts were made, as tolerated by the participant and while adequate cooperation could be maintained, to obtain three valid measurements. An acquisition was considered unsuccessful when the instrument failed to generate or display a valid measurement value. For LT and WTW, values recorded as 0 were considered to represent unsuccessful measurements and were therefore recoded as missing before statistical analysis. Measurements that did not satisfy the instrument’s built-in quality-control criteria, including those flagged because of excessive variability among repeated acquisitions, were repeated. If three valid measurements could not be obtained for a given parameter, that parameter was recorded as an unsuccessful measurement and was excluded from the corresponding analysis. Both devices were calibrated according to the manufacturers’ recommendations before measurement sessions.

Proportional bias was assessed by ordinary least-squares linear regression of the inter-device difference (Lenstar LS900 − Swan SW600) against the pairwise mean. A slope significantly different from zero was considered evidence of proportional bias. All analyses were based on parameter-specific complete measurement pairs without imputation.

### 2.4. Statistical Analysis

Statistical analyses were performed using SPSS software (version 27.0; Chicago, IL, USA). Results are presented as means ± standard deviation (SD) with 95% confidence intervals where appropriate. Within-session repeatability of Swan SW600 measurements was summarized using within-subject standard deviation (Sw), coefficient of repeatability (CoR = 1.96 × √2 × Sw), coefficient of variation (CoV = Sw/mean × 100%), and intraclass correlation coefficient (ICC). ICCs were calculated using a two-way mixed-effects model, absolute-agreement definition, and single-measurement form.

Between-device agreement was evaluated primarily using the paired mean difference, ICC, and Bland–Altman analysis with 95% limits of agreement (mean difference ± 1.96 SD). ICCs were also calculated using a two-way mixed-effects model with an absolute-agreement definition and a single-measurement type. The difference was defined as Lenstar LS900 minus Swan SW600. Paired-sample *t* tests assessed systematic differences between devices. Spearman correlation coefficients were calculated as secondary measures of association and were not interpreted as measures of agreement. Acquisition success rates between the two devices were compared using exact McNemar tests because measurements were paired within participants. A two-sided *p* value < 0.05 was considered statistically significant. Analyses were performed for the full sample and separately for age groups of 3–6, 7–11, and 12–18 years.

No imputation was performed for missing or unsuccessful measurements. Between-device agreement analyses were conducted using a parameter-specific complete-case approach. For each biometric parameter, only participants with valid measurements from both devices were included in the calculation of the paired mean difference, ICC, Bland–Altman limits of agreement and paired-sample *t* test. All agreement statistics for a given parameter were calculated using the same set of complete measurement pairs.

## 3. Results

### 3.1. Subjects

This study included a total of 778 preschool children to adolescents in Beijing from September 2024 to June 2025, comprising 399 boys (51.3%) and 379 girls (48.7%). The age ranged from 3 to 18 years, with an overall mean age of 7.45 ± 3.01 years, a mean age of 7.53 ± 3.06 years for boys, and a mean age of 7.37 ± 2.96 years for girls. The age-stratified groups included 322 participants aged 3–6 years, 373 aged 7–11 years, and 83 aged 12–18 years.

Additional bilateral analyses showed strong inter-ocular agreement for most biometric parameters measured with both devices. For the Swan SW600, the Spearman ρ/ICC values were 0.977/0.970 for AL, 0.937/0.761 for CCT, 0.933/0.925 for AD, 0.890/0.840 for LT, 0.973/0.977 for R1, 0.965/0.970 for R2, and 0.970/0.958 for AL/CR. WTW showed comparatively weaker inter-ocular agreement, with ρ = 0.710 and ICC = 0.308.

Similar findings were observed for the Lenstar LS900. The Spearman ρ/ICC values were 0.978/0.973 for AL, 0.893/0.863 for CCT, 0.942/0.933 for AD, 0.913/0.883 for LT, 0.956/0.962 for R1, 0.943/0.948 for R2, and 0.963/0.957 for AL/CR. WTW again showed comparatively weaker inter-ocular agreement, with ρ = 0.547 and ICC = 0.319.

### 3.2. Repeatability of Swan SW600 Measurements

A total of 778 participants were included in the repeatability analysis. The repeatability results of Swan SW600 measurements are summarized in Table 1. AL showed very high within-session repeatability (Sw = 0.0016 mm, CoR = 0.0043 mm, CoV = 0.0069%, ICC = 0.9999). R1, R2, CCT, AD, and AL/CR also showed ICC values above 0.99. Repeatability was lower for LT (ICC = 0.9060) and WTW (ICC = 0.8452), with WTW showing the largest CoV (6.876%).

### 3.3. Agreement Between Swan SW600 and Lenstar LS900

The number of valid measurements varied across parameters because valid acquisitions could not be obtained for every parameter in every participant, particularly for AD, LT, and WTW with the Lenstar LS900. Consequently, the sample size for each between-device agreement analysis was determined by the number of participants with valid measurements from both devices for the corresponding parameter.

**Figure 1:**
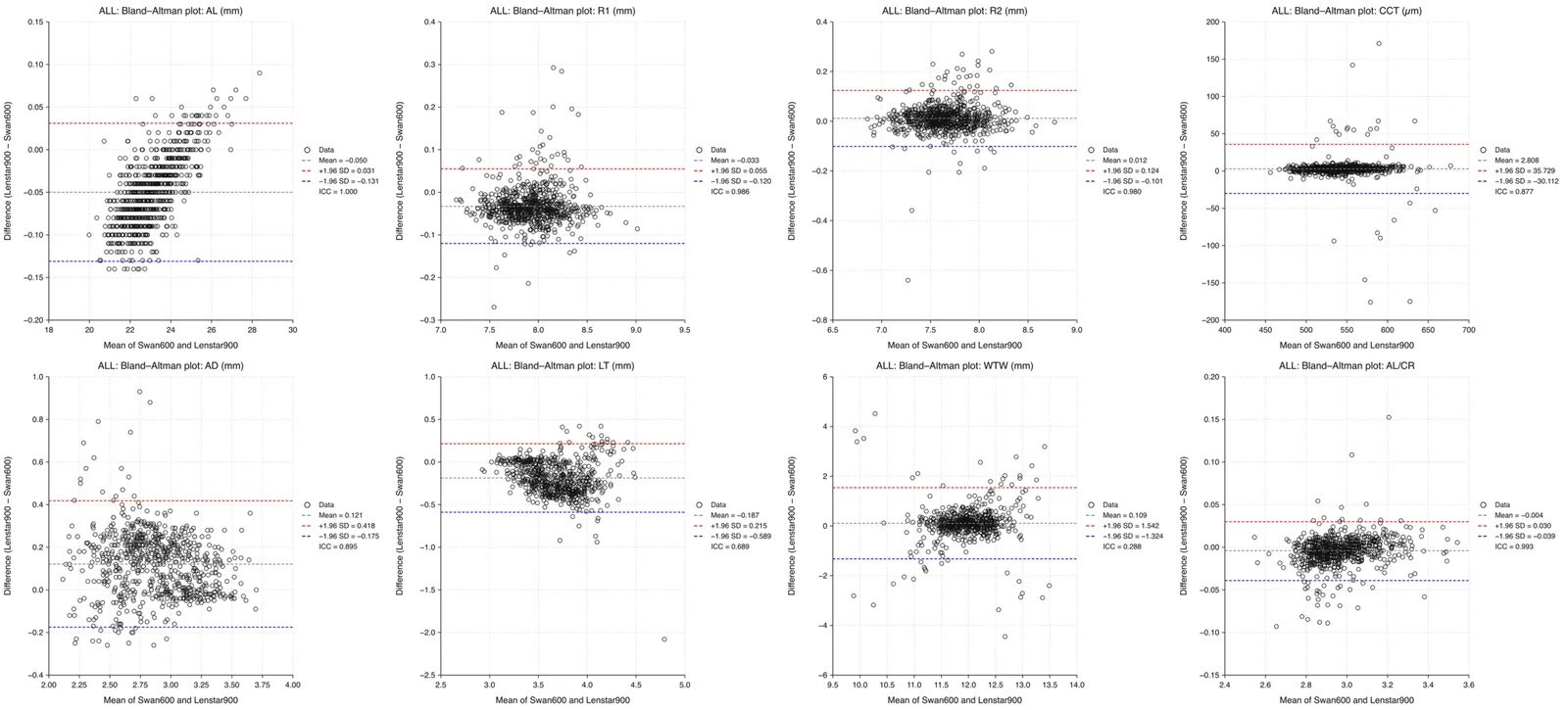
Bland–Altman plot of agreement between Lenstar LS900 and Swan SW600 across all age groups. Difference in the means was computed by subtracting SW600 values from LS900 values. The gray, red, and blue dashed lines represent the mean difference, upper 95% limit of agreement, and lower 95% limit of agreement, respectively. AL, Axial Length; R1, Radius in the flattest meridian; R2, Radius in the steepest meridian; CCT, Central Corneal Thickness; AD, Anterior Chamber Depth; LT, Lens Thickness; WTW, White-To-White distance; AL/CR, Axial Length/Corneal Radius. Difference in the means was computed by subtracting SW600 values from LS900 values; SD, Standard Deviation; ICC, Intraclass Correlation Coefficient.

Overall agreement is summarized in Table 2 and Figure 1. AL showed the closest agreement between devices: Swan SW600 measured 23.094 ± 1.216 mm and Lenstar LS900 measured 23.044 ± 1.245 mm, corresponding to a mean difference of −0.050 ± 0.041 mm (LS900 − SW600), 95% LoA of −0.131 to 0.031 mm, and ICC of 1.000. R1, R2, and AL/CR also showed high agreement. The mean differences for R1 and R2 were −0.033 ± 0.045 mm and 0.012 ± 0.057 mm, respectively, with ICC values of 0.986 and 0.980. The mean difference for AL/CR was −0.004 ± 0.018, with an ICC value of 0.993.

CCT and AD showed lower agreement than AL and corneal radii, with ICCs of 0.877 and 0.895, respectively. LT showed moderate inter-device agreement, with an ICC of 0.689, a mean difference of −0.187 ± 0.205 mm, and 95% LoA of −0.589 to 0.215 mm. WTW showed the weakest agreement among the evaluated parameters. The mean inter-device difference was 0.109 ± 0.731 mm, with a 95% CI of 0.051 to 0.167 mm and a wide 95% LoA of −1.324 to 1.542 mm. The Spearman correlation coefficient and ICC for WTW were 0.586 and 0.288, respectively. These results indicate substantially greater inter-device variability for WTW than for AL, corneal radii, and AL/CR. Although paired differences reached statistical significance, clinical comparability was interpreted primarily on the basis of the magnitude of the mean difference, 95% LoA, and ICC rather than statistical significance alone.

Because of slight deviations from normality, Spearman correlation coefficients were used to assess associations between devices. The strength of the correlations between measurements obtained with the Swan SW600 and Lenstar LS900 varied across parameters. The correlation coefficients were highest for AL (ρ = 1.00), followed by R1 (ρ = 0.99), R2 (ρ = 0.98), and AL/CR (ρ = 0.99). CCT and AD showed good correlations (ρ > 0.90), whereas LT showed moderate correlation (ρ = 0.83). WTW demonstrated a relatively weak correlation (ρ = 0.59).

### 3.4. Age-Stratified Agreement

**Figure 2:**
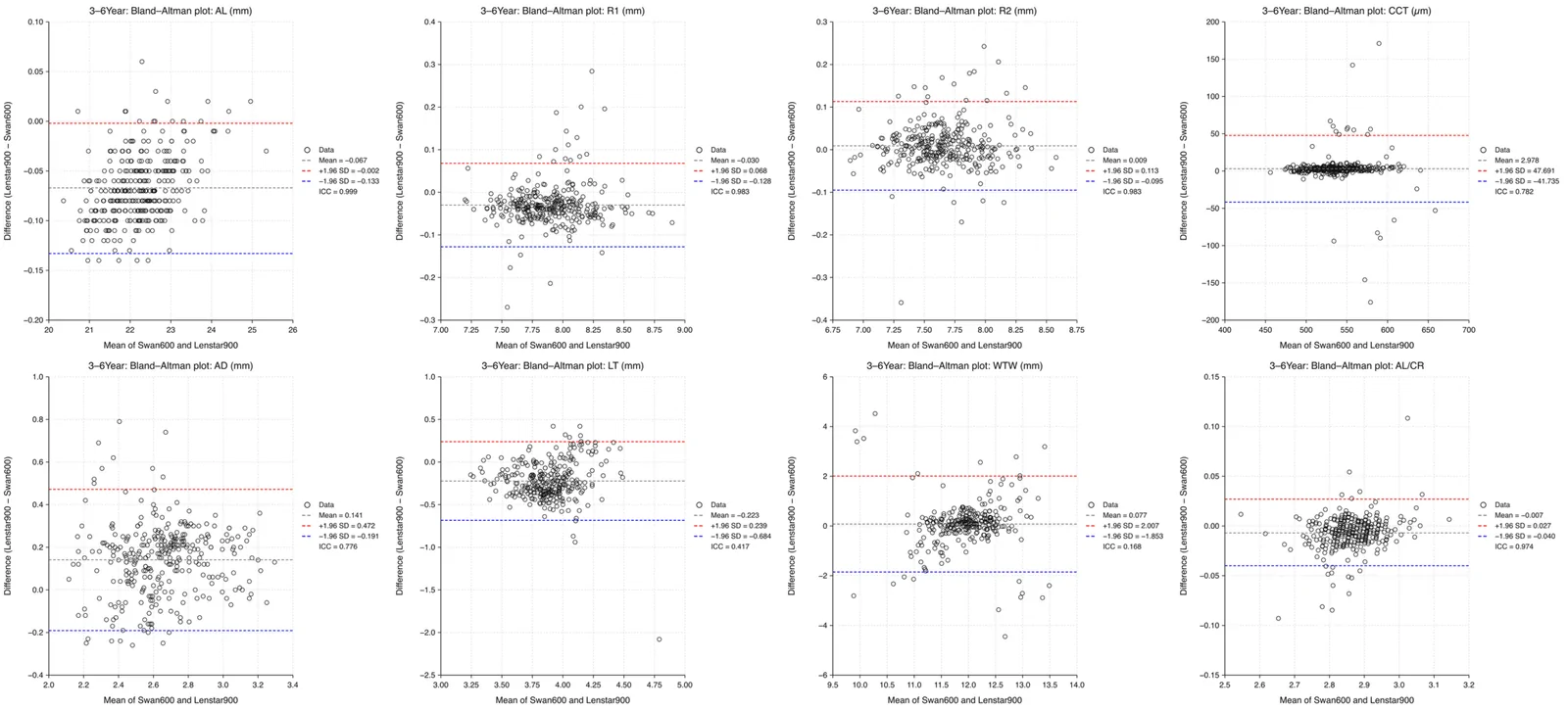
Bland–Altman plot of agreement between Lenstar LS900 and Swan SW600 in the 3–6-year group. Difference in the means was computed by subtracting SW600 values from LS900 values. The gray, red, and blue dashed lines represent the mean difference, upper 95% limit of agreement, and lower 95% limit of agreement, respectively. AL, Axial Length; R1, Radius in the flattest meridian; R2, Radius in the steepest meridian; CCT, Central Corneal Thickness; AD, Anterior Chamber Depth; LT, Lens Thickness; WTW, White-To-White distance; AL/CR, Axial Length/Corneal Radius; SD, Standard Deviation; ICC, Intraclass Correlation Coefficient.

**Figure 3:**
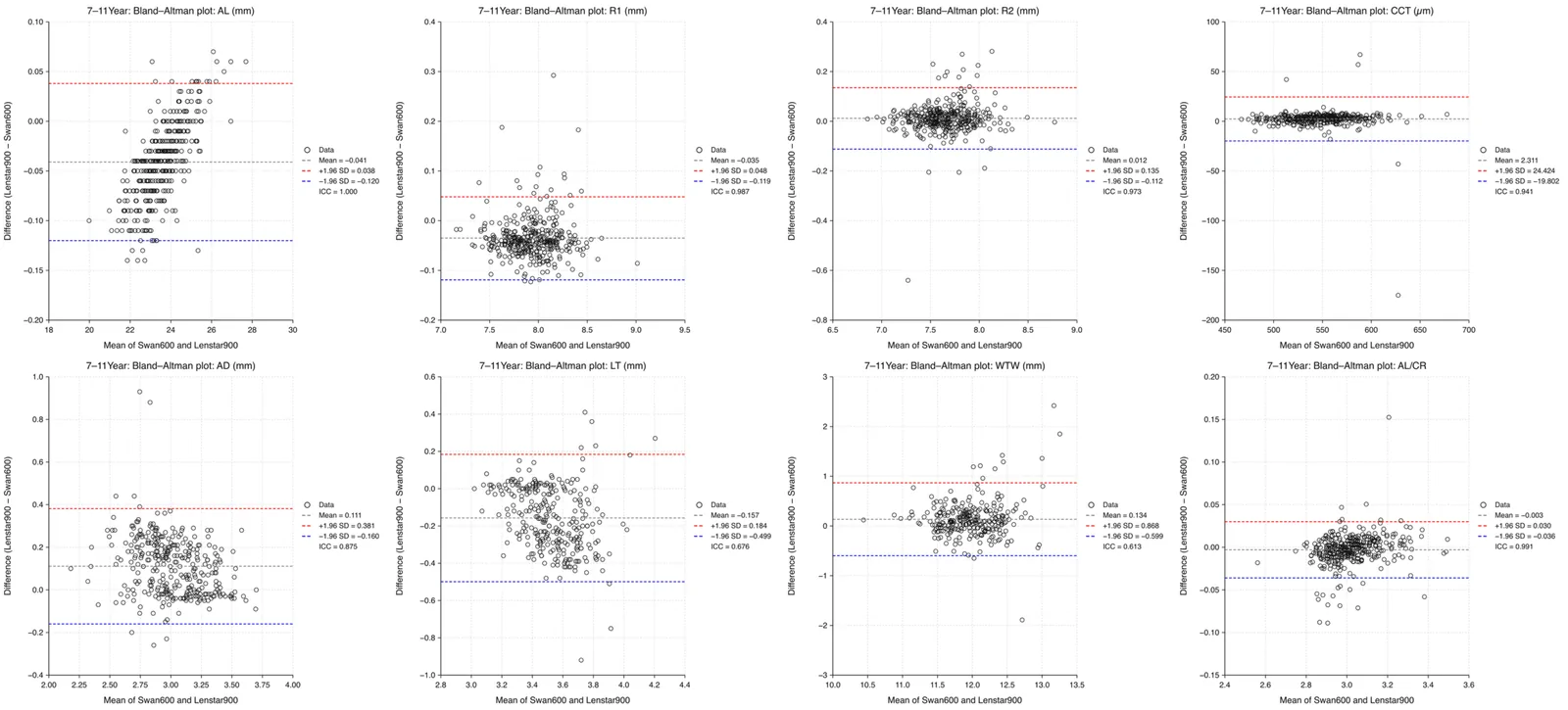
Bland–Altman plot of agreement between Lenstar LS900 and Swan SW600 in the 7–11-year group. Difference in the means was computed by subtracting SW600 values from LS900 values. The gray, red, and blue dashed lines represent the mean difference, upper 95% limit of agreement, and lower 95% limit of agreement, respectively. AL, Axial Length; R1, Radius in the flattest meridian; R2, Radius in the steepest meridian; CCT, Central Corneal Thickness; AD, Anterior Chamber Depth; LT, Lens Thickness; WTW, White-To-White distance; AL/CR, Axial Length/Corneal Radius; SD, Standard Deviation; ICC, Intraclass Correlation Coefficient.

**Figure 4:**
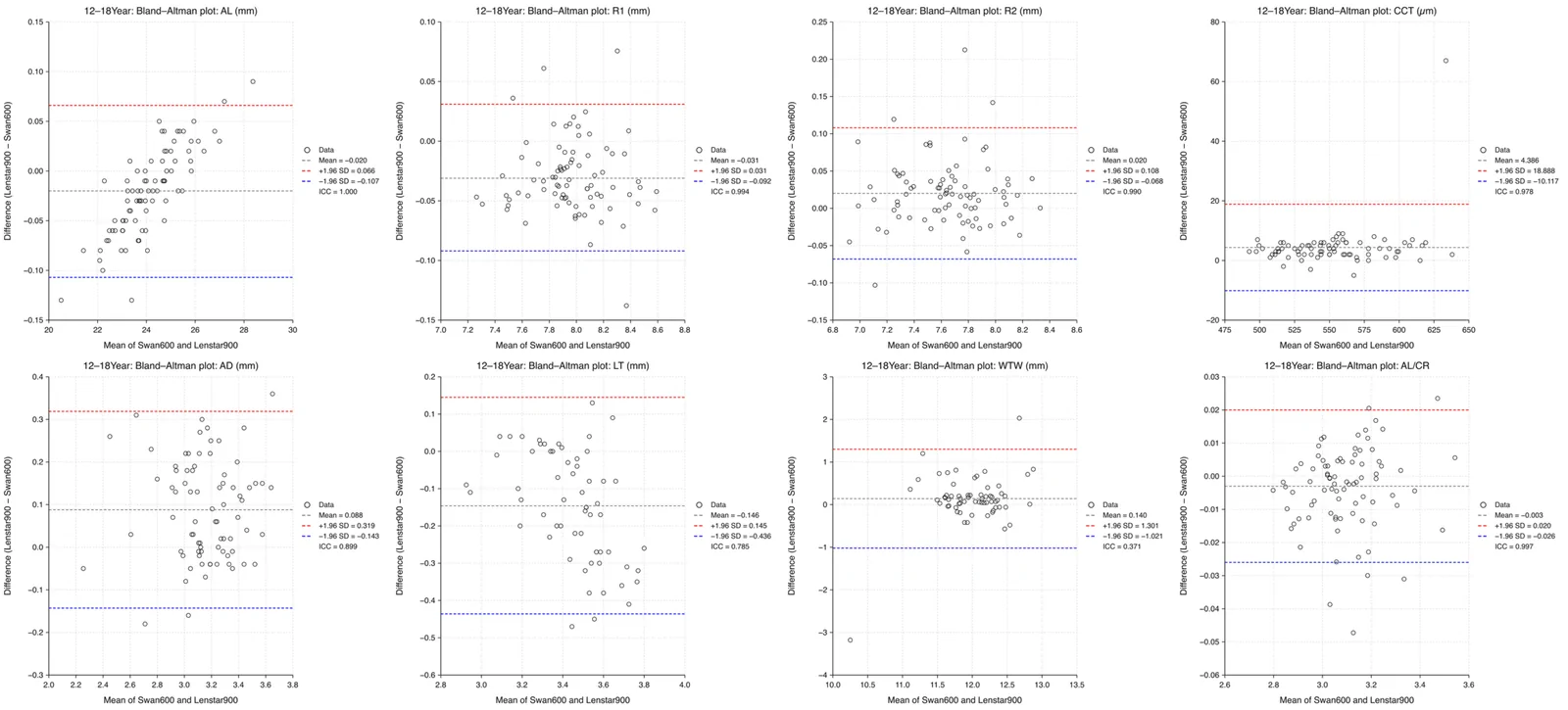
Bland–Altman plot of agreement between Lenstar LS900 and Swan SW600 in the 12–18-year group. Difference in the means was computed by subtracting SW600 values from LS900 values. The gray, red, and blue dashed lines represent the mean difference, upper 95% limit of agreement, and lower 95% limit of agreement, respectively. AL, Axial Length; R1, Radius in the flattest meridian; R2, Radius in the steepest meridian; CCT, Central Corneal Thickness; AD, Anterior Chamber Depth; LT, Lens Thickness; WTW, White-To-White distance; AL/CR, Axial Length/Corneal Radius; SD, Standard Deviation; ICC, Intraclass Correlation Coefficient.

Age-stratified results are summarized in Table 3, Table 4 and Table 5 and Figure 2, Figure 3 and Figure 4. Agreement for AL remained high in all age groups, with ICC = 0.999 in children aged 3–6 years and ICC = 1.000 in both older groups. The AL mean difference decreased in magnitude from −0.067 mm in the 3–6-year group to −0.041 mm in the 7–11-year group and −0.020 mm in the 12–18-year group. R1, R2, and AL/CR also retained high ICC values across all three age strata.

Agreement for several anterior-segment parameters was more variable across age groups. In children aged 3–6 years, the ICCs were 0.782 for CCT, 0.776 for AD, 0.417 for LT, and 0.168 for WTW. Agreement for CCT, AD, and LT generally improved with increasing age, with ICCs of 0.941, 0.875, and 0.676, respectively, in the 7–11-year group and 0.978, 0.899, and 0.785, respectively, in the 12–18-year group. In contrast, WTW agreement showed greater variability across age strata, with ICCs of 0.168, 0.613, and 0.371 in the 3–6-year, 7–11-year, and 12–18-year groups, respectively. These findings indicate that age-stratified agreement was parameter-specific, with consistently high agreement for AL, R1, R2, and AL/CR but more variable agreement for anterior-segment parameters, particularly WTW.

The Swan SW600 yielded a higher proportion of valid measurements than the Lenstar LS900 for AD, LT, and WTW. In the overall population, acquisition success rates for AD, LT, and WTW were 100.0% (778/778), 100.0% (778/778), and 98.3% (765/778), respectively, with the Swan SW600, compared with 94.9% (738/778), 84.3% (656/778), and 80.6% (627/778), respectively, with the Lenstar LS900. Paired comparisons using exact McNemar tests showed significantly higher success rates with the Swan SW600 for all three parameters (all *p* < 0.01).

Age-stratified analyses showed a generally similar pattern. In children aged 3–6 years, valid AD, LT, and WTW measurements were obtained in 100.0%, 100.0%, and 97.8% of participants with the Swan SW600, compared with 99.1%, 94.1%, and 87.9% with the Lenstar LS900, respectively. The differences were significant for LT and WTW (both *p* < 0.01), but not for AD (*p* = 0.250). In the 7–11-year group, the corresponding success rates were 100.0%, 100.0%, and 99.2% with the Swan SW600 versus 92.0%, 78.6%, and 74.5% with the Lenstar LS900, respectively; all three differences were significant (all *p* < 0.01). Among participants aged 12–18 years, the corresponding success rates were 100.0%, 100.0%, and 96.4% with the Swan SW600 versus 91.6%, 72.3%, and 79.5% with the Lenstar LS900, respectively. The Swan SW600 showed significantly higher acquisition success rates for AD (*p* < 0.05), LT (*p* < 0.01), and WTW (*p* < 0.01).

### 3.5. Proportional-Bias Analysis

In the overall population, significant proportional bias was detected for AL (β = 0.0235; *p* < 0.001; R^2^ = 0.491), AD (β = −0.0737; *p* < 0.001; R^2^ = 0.024), LT (β = −0.1420; R^2^ = 0.038), and AL/CR (β = 0.0288; *p* < 0.001; R^2^ = 0.053). A small positive regression slope was also observed for R2 (β = 0.0154; *p* = 0.035; R^2^ = 0.006), whereas no significant proportional bias was detected for R1, CCT, or WTW.

For LT, no significant proportional bias was detected in children aged 3–6 years (β = 0.0248; *p* = 0.683), whereas significant negative proportional bias was observed in the 7–11-year (β = −0.2575; *p* < 0.001; R^2^ = 0.081) and 12–18-year groups (β = −0.3642; *p* < 0.001; R^2^ = 0.226).

For WTW, no significant proportional bias was detected in the overall population (β = 0.0909; *p* = 0.128; R^2^ = 0.004) or in children aged 3–6 years (β = 0.0198; *p* = 0.846). In the exploratory age-stratified analyses, significant positive slopes were observed in the 7–11-year (β = 0.1360; *p* = 0.016; R^2^ = 0.021) and 12–18-year groups (β = 0.4619; *p* = 0.006; R^2^ = 0.119).

## 4. Discussion

The present study evaluated the repeatability of the Swan SW600 and its agreement with the Lenstar LS900 for ocular biometric measurements in a large pediatric population ranging from preschool-aged children to adolescents. The main findings were that the Swan SW600 showed high repeatability and close agreement with the Lenstar LS900 for AL, corneal radii, and AL/CR. Importantly, this high level of agreement was maintained in children aged 3–6 years. In contrast, agreement was weaker for lens thickness (LT) and white-to-white distance (WTW). In addition, the Swan SW600 yielded a higher proportion of valid measurements for anterior-segment parameters such as AD, LT, and WTW. These findings support the potential clinical utility of the Swan SW600 for pediatric ocular biometry, particularly for axial and corneal measurements.

AL is one of the most important structural parameters for monitoring ocular growth and myopia progression in children. Previous studies have demonstrated high repeatability and agreement among established optical biometers for AL measurements [22,23].

In the present study, AL measurements obtained with the Swan SW600 showed strong statistical agreement with those obtained using the Lenstar LS900, with a mean inter-device difference of −0.050 mm and 95% limits of agreement from −0.131 to 0.031 mm. The small mean bias and high ICC indicate a high level of consistency between the two devices for AL measurement at the group level. However, the limits of agreement indicate that the difference between devices for an individual child may exceed 0.10 mm. Therefore, these findings should not be interpreted as demonstrating clinical interchangeability. No predefined clinically acceptable limits of agreement were established in the present study, and there is currently no universally accepted threshold for clinically acceptable inter-device AL differences in children. Furthermore, without an independent reference standard, the present study cannot determine which device provides the more accurate AL measurement when discrepancies occur. Therefore, for longitudinal monitoring of axial elongation, particularly during myopia-control follow-up, the same biometer should preferably be used at successive visits to minimize the potential influence of inter-device measurement differences.

The favorable repeatability observed for AL may partly relate to the optical and acquisition design of the Swan SW600. According to the manufacturer’s technical specifications, the device uses a broadband superluminescent diode centered at approximately 850 nm with a spectral bandwidth of approximately 100 nm. A broader spectral bandwidth shortens the coherence length and produces a narrower interference envelope, which theoretically facilitates more precise localization of reflecting ocular interfaces. The device also incorporates a high-speed reference-arm design and high-frequency signal acquisition, allowing a large amount of interferometric information to be collected within a short acquisition period. These features may help reduce the influence of individual acquisition noise and may contribute to the high within-device repeatability observed for AL in the present study.

At the same time, high within-device repeatability does not necessarily imply the absence of systematic differences between devices. A significant positive proportional bias was observed for AL, indicating that the inter-device difference varied with ocular length. Although both instruments are based on OLCR, they differ in illumination wavelength, optical configuration, signal acquisition and processing. Small systematic differences in these processes may become more apparent across longer optical paths and could partly contribute to the magnitude-dependent difference observed for AL. Because the detailed signal-processing and refractive-index algorithms of the Swan SW600 are proprietary, the specific mechanism cannot be determined from the present data.

Similarly, excellent agreement was observed for corneal radii and AL/CR. The AL/CR ratio integrates axial elongation and corneal curvature and has been shown to be strongly associated with refractive status and myopia in children [13]. In the present study, both R1 and R2 showed high inter-device agreement, and AL/CR demonstrated similarly strong agreement. These findings are important because relatively small discrepancies in either AL or corneal curvature may influence the calculated AL/CR ratio. The close agreement observed for both components resulted in highly consistent AL/CR measurements between the two devices, supporting the potential use of the Swan SW600 for assessing refractive development in children.

A particularly relevant finding from a pediatric perspective was the preservation of high agreement for AL, corneal radii, and AL/CR in preschool-aged children. Children aged 3–6 years often have greater difficulty maintaining stable fixation and cooperating with prolonged ophthalmic examinations. Despite these potential challenges, agreement for the principal axial and corneal parameters remained excellent in this age group. In contrast, agreement for several anterior-segment parameters was lower in younger children. CCT and AD showed moderate-to-good agreement, whereas LT and WTW demonstrated substantially greater variability. This pattern suggests that age and cooperation may influence some biometric measurements more strongly than AL or corneal curvature. Nevertheless, because participant cooperation and fixation stability were not quantitatively assessed in the present study, the contribution of these factors cannot be determined directly.

In the present study, CCT showed generally good agreement, whereas AD demonstrated a modest systematic difference between devices [16,18]. The lower agreement observed for some anterior-segment parameters may partly reflect their greater dependence on precise localization of closely spaced anatomical interfaces. Small differences in the identification of the anterior and posterior corneal or lens surfaces, the reference planes used for measurement, signal processing, or refractive-index conversion may translate into measurable inter-device offsets.

LT showed weaker agreement, indicating that measurements obtained using the two devices should not be assumed to be directly interchangeable. LT may be particularly sensitive to these factors because its measurement requires accurate identification of both the anterior and posterior lens surfaces. In addition, lens thickness and configuration vary with accommodation, particularly in children who generally have strong accommodative responses.

Furthermore, significant proportional bias was observed for LT, with the LS900-minus-SW600 difference becoming more negative as LT increased. This magnitude-dependent difference indicates that the mean bias alone does not fully characterize agreement and that a single fixed correction may not adequately account for inter-device differences across the LT measurement range. One possible explanation is that small differences in lens-boundary localization or optical-path conversion become more apparent as lens thickness increases. This interpretation remains speculative, however, because the detailed boundary-detection and refractive-index algorithms used by the Swan SW600 are not publicly available.

WTW showed substantially weaker inter-device agreement than AL and corneal radius measurements. Although the mean inter-device difference was relatively small, the wide 95% limits of agreement indicate considerable variability at the individual level. In the 12–18-year subgroup, the mean difference was 0.140 mm, whereas the 95% limits of agreement ranged from −1.021 to 1.301 mm and the ICC was 0.371. Thus, the small average bias should not be interpreted as evidence of clinical interchangeability.

The relatively poor agreement for WTW may also reflect the nature of the anatomical landmark being measured. Unlike axial interferometric measurements, WTW depends on identification of the corneoscleral or limbal boundary, which represents a gradual transition rather than a sharply defined optical interface. The detected boundary may therefore be sensitive to image contrast, illumination, segmentation threshold, ocular centration, eyelid opening, and head or eye position. Differences in image processing and limbal-edge identification between devices could consequently produce substantial individual-level variability even when the average inter-device bias is small.

No significant proportional bias was detected for WTW in the overall population, although exploratory age-stratified analyses suggested magnitude-dependent differences in the older age groups. These findings suggest that WTW measurements obtained with the Swan SW600 and Lenstar LS900 should be interpreted cautiously when compared at the individual level or during longitudinal follow-up.

Taken together, the parameter-specific differences observed in this study are likely to reflect a combination of device- and participant-related factors rather than a single source of error. Differences in optical configuration, signal processing, anatomical boundary identification, and refractive-index assumptions may contribute to systematic inter-device differences, whereas ocular surface conditions, fixation, accommodation, participant cooperation, and head or eye positioning may introduce additional measurement variability. Because these factors were not independently quantified and several technical details of the Swan SW600 remain proprietary, their individual contributions cannot be determined from the present data.

Another noteworthy finding was the higher proportion of valid anterior-segment measurements obtained with the Swan SW600. Overall, acquisition success rates for AD, LT, and WTW were significantly higher with the Swan SW600 than with the Lenstar LS900 (all *p* < 0.01). This pattern was generally maintained across age groups, although the difference in AD was not significant among children aged 3–6 years. The advantage was particularly evident for LT and WTW. In addition to this higher measurement success rate, our practical experience during the study suggested that examinations could generally be completed more rapidly with the Swan SW600. When both eyes were measured approximately three times in preschool-aged children, the examination typically required about 2–3 min with the Swan SW600 compared with approximately 4–7 min with the Lenstar LS900. These observations were not based on prospectively or systematically recorded examination times and should therefore be regarded as exploratory.

The higher acquisition efficiency may partly relate to the hardware and fixation design of the Swan SW600. According to the manufacturer, its high-speed reference-arm scanning and high-frequency signal acquisition allow multiple interferometric signals to be acquired over a relatively short period. A shorter acquisition sequence may reduce the duration for which stable fixation and head position must be maintained, which may be particularly helpful in younger children. The built-in audiovisual fixation system may provide an additional advantage by helping to maintain attention during measurement. These features could contribute to the higher acquisition success rates observed in this pediatric cohort, although fixation stability, participant movement, and the number and duration of individual acquisition attempts were not objectively recorded.

There may also be practical differences between the two instruments beyond measurement success. In the configuration used at our center, the Swan SW600 incorporates an integrated display and has a relatively compact setup, whereas the Lenstar LS900 was used with an external display. A more compact configuration may reduce space requirements and facilitate transportation and deployment outside specialized ophthalmic clinics. These features could be useful in school-based screening, community programs, and primary-care settings, where examination space and equipment transport may be important considerations.

Initial equipment cost may represent another practical factor in large-scale implementation. The Swan SW600 also has a substantially lower acquisition cost than the Lenstar LS900 in the current domestic market. Although equipment cost was not formally evaluated in the present study, a lower initial purchase cost, together with the compact configuration and potentially shorter examination time, may reduce barriers to implementation in high-volume or resource-limited pediatric settings.

These considerations may be particularly relevant in China, where pediatric myopia screening involves very large numbers of preschool- and school-aged children. At this scale, even modest reductions in examination time, equipment requirements, transportation burden, or staffing time could improve screening capacity and reduce practical barriers to extending ocular biometry to community and primary-care settings. However, these potential organizational and economic advantages remain to be tested in formal workflow and health-economic studies.

Importantly, the higher measurement success rate did not necessarily correspond to stronger inter-device agreement. Although LT and WTW measurements were obtained more frequently with the Swan SW600, agreement with the Lenstar LS900 was weaker for these parameters, particularly for WTW. Measurement feasibility and inter-device agreement therefore represent distinct aspects of device performance. From the perspective of pediatric myopia screening, however, the principal biometric parameters of interest are generally AL, corneal radius, and the derived AL/CR ratio, all of which demonstrated high repeatability and close agreement between the two devices in the present study, including in preschool-aged children. For pediatric myopia-screening applications that primarily rely on AL and AL/CR, the weaker agreement observed for LT and WTW may be less consequential; however, these parameters remain relevant for comprehensive ocular biometry and should be interpreted cautiously when measurements from different devices are compared.

Taken together, the combination of reliable measurements for key myopia-related parameters, a high rate of successful acquisition, and potentially shorter examination times suggests that the Swan SW600 may have practical clinical value in pediatric screening. Nevertheless, LT and particularly WTW measurements should not be considered interchangeable with those obtained using the Lenstar LS900 without further validation. Prospective studies incorporating standardized measurement of examination time, the number of acquisition attempts, and objective assessments of fixation and cooperation are warranted to confirm these potential operational advantages.

The present study has several strengths. First, the relatively large sample of 778 children and adolescents allowed the performance of the Swan SW600 to be evaluated across a broad pediatric age range. Second, the inclusion of 322 children aged 3–6 years enabled specific evaluation of device performance in preschool-aged children, a population in whom ocular biometry may be more technically challenging. Third, several ocular biometric parameters were assessed simultaneously, allowing the performance of the device to be characterized comprehensively rather than on the basis of AL alone. The age-stratified analyses further revealed parameter- and age-group-specific patterns of agreement, providing clinically relevant information regarding the potential application of the Swan SW600 in pediatric populations.

Several limitations should also be acknowledged. First, this was a single-center study conducted in healthy Chinese children and adolescents; therefore, the findings may not be directly generalizable to children from other populations or to patients with ocular abnormalities. In particular, the repeatability and inter-device agreement observed in the present study cannot be directly extrapolated to children with keratoconus, cataract, high myopia with posterior staphyloma, or other ocular pathologies that may affect ocular anatomy, optical media, fixation, or measurement quality. Further studies in these clinical populations are required. Second, although bilateral measurements were obtained and most biometric parameters showed strong inter-ocular correlation and absolute agreement, only the left eye was included in the primary analysis to preserve statistical independence. Inter-ocular agreement was not uniformly high across all parameters, with notably weaker agreement for WTW; therefore, potential eye-specific differences cannot be completely excluded when generalizing the findings to the fellow eye. Third, the present study assessed only within-session repeatability; between-session test–retest repeatability was not evaluated. Fourth, examination time, fixation quality, participant cooperation, and the number of repeated measurement attempts were not prospectively or systematically recorded. Consequently, although the Swan SW600 produced a higher proportion of valid measurements for several parameters, the mechanism underlying this difference cannot be established. Moreover, the perceived advantages of shorter examination time and audiovisual fixation guidance represent anecdotal observations rather than formally evaluated outcomes and require prospective validation using standardized objective assessments. Fifth, detailed information regarding the proprietary measurement algorithms and refractive indices used by the Swan SW600 was unavailable, limiting our ability to explain the observed systematic differences between devices. Sixth, the 12–18-year subgroup was relatively small compared with the younger age groups, and the number of complete measurement pairs was further reduced for some parameters, particularly LT and WTW. Therefore, subgroup-specific agreement estimates in this age group may have lower statistical precision and should be interpreted cautiously. Finally, the present study assessed repeatability and cross-sectional inter-device agreement and therefore cannot determine whether the Swan SW600 can reliably detect true longitudinal changes in AL over time. Dedicated longitudinal studies incorporating repeated measurements across separate visits are required to determine the reproducibility and sensitivity of the Swan SW600 for detecting axial elongation and tracking myopia progression.

Overall, the present findings indicate that the Swan SW600 provides highly repeatable measurements and close agreement with the Lenstar LS900 for AL, corneal radii, and AL/CR in children and adolescents, including preschool-aged children. Its high acquisition success, relatively rapid examination, and compact integrated configuration may offer practical advantages in pediatric and high-volume screening settings. However, agreement was weaker for LT and particularly WTW, indicating that these measurements should be interpreted cautiously and should not automatically be considered interchangeable with those obtained using the Lenstar LS900. Further studies incorporating direct assessments of acquisition time, fixation and cooperation, measurement success, workflow efficiency, cost-effectiveness, and longitudinal reproducibility are warranted to clarify the clinical utility of the Swan SW600 in pediatric ophthalmic screening and follow-up.

## Figures and Tables

**Table 1:** Repeatability of Swan SW600.

**Parameter**	**Sw**	**CoR**	**CoV (%)**	**ICC**
AL (mm)	0.0016	0.0043	0.0069	0.9999
R1 (mm)	0.0070	0.0195	0.0882	0.9993
R2 (mm)	0.0035	0.0096	0.0458	0.9998
CCT (µm)	3.1200	8.6410	0.5740	0.9918
AD (mm)	0.0050	0.0140	0.1780	0.9998
LT (mm)	0.1150	0.3184	3.0400	0.9060
WTW (mm)	0.8045	2.2284	6.8760	0.8452
AL/CR	0.0019	0.0052	0.0636	0.9998

AL, axial length; R1, radius in the flattest meridian; R2, radius in the steepest meridian; CCT, central corneal thickness; AD, anterior chamber depth; LT, lens thickness; WTW, white-to-white distance; AL/CR, axial length/corneal radius ratio; Sw, within-subject standard deviation; CoR, coefficient of repeatability; CoV, coefficient of variation; ICC, intraclass correlation coefficient.

**Table 2:** Agreement of ocular biometric parameters between Swan SW600 and Lenstar LS900.

**Parameter**	**Device**	**Valid N**	**Paired N**	**Mean ± SD**	**Range (Minimum–Maximum)**	**Mean Difference ± SD**	**95% CI of Difference**	**95% LoA**	**ρ**	**ICC**	**Paired-Samples** ***t*** **Test (t)**	***p*** **Value**
AL (mm)	SW600	778	778	23.094 ± 1.216	20.030–28.320	−0.050 ± 0.041	−0.053 to −0.047	−0.131 to 0.031	1.000	1.000	−33.576	<0.01
	LS900	778		23.044 ± 1.245	19.930–28.410							
R1 (mm)	SW600	778	778	7.938 ± 0.267	7.168–9.056	−0.033 ± 0.045	−0.036 to −0.029	−0.120 to 0.055	0.986	0.986	−20.291	<0.01
	LS900	778		7.906 ± 0.269	7.150–8.970							
R2 (mm)	SW600	778	778	7.646 ± 0.281	6.851–8.773	0.012 ± 0.057	0.007 to 0.016	−0.101 to 0.124	0.981	0.980	5.588	<0.01
	LS900	778		7.658 ± 0.286	6.860–8.770							
CCT (µm)	SW600	778	778	543.560 ± 34.151	457.000–715.000	2.808 ± 16.796	1.628 to 3.989	−30.112 to 35.729	0.933	0.877	4.664	<0.01
	LS900	778		546.369 ± 33.563	455.000–681.000							
AD (mm)	SW600	778	738	2.809 ± 0.338	1.940–3.740	0.121 ± 0.151	0.110 to 0.132	−0.175 to 0.418	0.903	0.895	21.762	<0.01
	LS900	738		2.930 ± 0.315	2.090–3.830							
LT (mm)	SW600	778	656	3.766 ± 0.317	2.970–5.830	−0.187 ± 0.205	−0.203 to −0.171	−0.589 to 0.215	0.826	0.689	−23.293	<0.01
	LS900	656		3.591 ± 0.280	2.880–4.550							
WTW (mm)	SW600	765	615	11.918 ± 0.587	7.830–14.900	0.109 ± 0.731	0.051 to 0.167	−1.324 to 1.542	0.586	0.288	3.696	<0.01
	LS900	627		12.026 ± 0.630	8.480–15.000							
AL/CR	SW600	778	778	2.967 ± 0.139	2.540–3.540	−0.004 ± 0.018	−0.006 to −0.003	−0.039 to 0.030	0.990	0.993	−7.044	<0.01
	LS900	778		2.962 ± 0.143	2.552–3.546							

SW600, Swan SW600; LS900, Lenstar LS900; AL, Axial Length; R1, Radius in the flattest meridian; R2, Radius in the steepest meridian; CCT, Central Corneal Thickness; AD, Anterior Chamber Depth; LT, Lens Thickness; WTW, White-To-White distance; AL/CR, Axial Length/Corneal Radius. Difference in the means was computed by subtracting SW600 values from LS900 values; SD, Standard deviation; CI, Confidence interval; LoA, Limits of agreement; ρ, Spearman correlation coefficient; ICC, Intraclass Correlation Coefficient; *p* value, paired-samples *t* test. Valid N, the number of participants with valid measurements obtained by each device. Paired N, the number of participants with valid measurements from both devices for the corresponding parameter.

**Table 3:** Agreement of ocular biometric parameters between Swan SW600 and Lenstar LS900 in children aged 3–6 years.

**Parameter**	**Device**	**Valid N**	**Paired N**	**Mean ± SD**	**Range (Minimum–Maximum)**	**Mean Difference ± SD**	**95% CI of Difference**	**95% LoA**	**ρ**	**ICC**	**Paired-Samples** ***t*** **Test (t)**	***p*** **Value**
AL (mm)	SW600	322	322	22.278 ± 0.772	20.400–25.360	−0.067 ± 0.034	−0.071 to −0.064	−0.133 to −0.002	0.999	0.999	−35.995	<0.01
	LS900	322		22.210 ± 0.785	20.320–25.330							
R1 (mm)	SW600	322	322	7.912 ± 0.270	7.194–8.931	−0.030 ± 0.050	−0.035 to −0.025	−0.128 to 0.068	0.984	0.983	−10.796	<0.01
	LS900	322		7.882 ± 0.274	7.190–8.860							
R2 (mm)	SW600	322	322	7.650 ± 0.290	6.905–8.588	0.009 ± 0.053	0.003 to 0.015	−0.095 to 0.113	0.985	0.983	3.090	<0.01
	LS900	322		7.659 ± 0.293	6.880–8.570							
CCT (µm)	SW600	322	322	538.298 ± 34.999	457.000–685.000	2.978 ± 22.813	0.486 to 5.470	−41.735 to 47.691	0.866	0.782	2.343	<0.05
	LS900	322		541.276 ± 33.963	455.000–675.000							
AD (mm)	SW600	322	319	2.578 ± 0.248	1.940–3.280	0.141 ± 0.169	0.122 to 0.159	−0.191 to 0.472	0.757	0.776	14.843	<0.01
	LS900	319		2.719 ± 0.256	2.090–3.390							
LT (mm)	SW600	322	303	4.005 ± 0.248	3.320–5.830	−0.223 ± 0.235	−0.249 to −0.196	−0.684 to 0.239	0.612	0.417	−16.440	<0.01
	LS900	303		3.778 ± 0.255	3.170–4.550							
WTW (mm)	SW600	315	277	11.913 ± 0.760	7.830–14.900	0.077 ± 0.985	−0.040 to 0.193	−1.853 to 2.007	0.457	0.168	1.296	<0.05
	LS900	283		11.997 ± 0.762	8.480–15.000							
AL/CR	SW600	322	322	2.866 ± 0.071	2.540–3.140	−0.007 ± 0.017	−0.008 to −0.005	−0.040 to 0.027	0.970	0.974	−6.875	<0.01
	LS900	322		2.859 ± 0.075	2.552–3.147							

SW600, Swan SW600; LS900, Lenstar LS900; AL, Axial Length; R1, Radius in the flattest meridian; R2, Radius in the steepest meridian; CCT, Central Corneal Thickness; AD, Anterior Chamber Depth; LT, Lens Thickness; WTW, White-To-White distance; AL/CR, Axial Length/Corneal Radius. Difference in the means was computed by subtracting SW600 values from LS900 values; SD, Standard deviation; CI, Confidence interval; LoA, Limits of agreement; ρ, Spearman correlation coefficient; ICC, Intraclass Correlation Coefficient; *p* value, paired-samples *t* test. Valid N, the number of participants with valid measurements obtained by each device. Paired N, the number of participants with valid measurements from both devices for the corresponding parameter.

**Table 4:** Agreement of ocular biometric parameters between Swan SW600 and Lenstar LS900 in children aged 7–11 years.

**Parameter**	**Device**	**Valid N**	**Paired N**	**Mean ± SD**	**Range (Minimum–Maximum)**	**Mean Difference ± SD**	**95% CI of Difference**	**95% LoA**	**ρ**	**ICC**	**Paired-Samples** ***t*** **Test (t)**	***p*** **Value**
AL (mm)	SW600	373	373	23.565 ± 1.068	20.030–27.660	−0.041 ± 0.040	−0.045 to −0.037	−0.120 to 0.038	0.999	1.000	−19.632	<0.01
	LS900	373		23.524 ± 1.097	19.930–27.720							
R1 (mm)	SW600	373	373	7.952 ± 0.259	7.168–9.056	−0.035 ± 0.043	−0.040 to −0.031	−0.119 to 0.048	0.986	0.987	−15.980	<0.01
	LS900	373		7.917 ± 0.259	7.150–8.970							
R2 (mm)	SW600	373	373	7.646 ± 0.265	6.851–8.773	0.012 ± 0.063	0.005 to 0.018	−0.112 to 0.135	0.975	0.973	3.558	<0.01
	LS900	373		7.658 ± 0.271	6.860–8.770							
CCT (µm)	SW600	373	373	546.794 ± 33.128	466.000–715.000	2.311 ± 11.282	1.166 to 3.456	−19.802 to 24.424	0.981	0.941	3.956	<0.01
	LS900	373		549.105 ± 32.336	468.000–681.000							
AD (mm)	SW600	373	343	2.955 ± 0.286	2.130–3.740	0.111 ± 0.138	0.096 to 0.125	−0.160 to 0.381	0.871	0.875	14.845	<0.01
	LS900	343		3.065 ± 0.247	2.230–3.720							
LT (mm)	SW600	373	293	3.601 ± 0.232	3.020–4.290	−0.157 ± 0.174	−0.177 to −0.137	−0.499 to 0.184	0.692	0.676	−15.475	<0.01
	LS900	293		3.443 ± 0.188	3.010–4.340							
WTW (mm)	SW600	370	275	11.919 ± 0.422	10.380–13.660	0.134 ± 0.374	0.090 to 0.179	−0.599 to 0.868	0.726	0.613	5.956	<0.01
	LS900	278		12.042 ± 0.468	10.500–14.380							
AL/CR	SW600	373	373	3.025 ± 0.123	2.570–3.490	−0.003 ± 0.017	−0.005 to −0.001	−0.036 to 0.030	0.988	0.991	−3.654	<0.01
	LS900	373		3.022 ± 0.127	2.552–3.499							

SW600, Swan SW600; LS900, Lenstar LS900; AL, Axial Length; R1, Radius in the flattest meridian; R2, Radius in the steepest meridian; CCT, Central Corneal Thickness; AD, Anterior Chamber Depth; LT, Lens Thickness; WTW, White-To-White distance; AL/CR, Axial Length/Corneal Radius. Difference in the means was computed by subtracting SW600 values from LS900 values; SD, Standard deviation; CI, Confidence interval; LoA, Limits of agreement; ρ, Spearman correlation coefficient; ICC, Intraclass Correlation Coefficient; *p* value, paired-samples *t* test. Valid N, the number of participants with valid measurements obtained by each device. Paired N, the number of participants with valid measurements from both devices for the corresponding parameter.

**Table 5:** Agreement of ocular biometric parameters between Swan SW600 and Lenstar LS900 in children aged 12–18 years.

**Parameter**	**Device**	**Valid N**	**Paired N**	**Mean ± SD**	**Range (Minimum–Maximum)**	**Mean Difference ± SD**	**95% CI of Difference**	**95% LoA**	**ρ**	**ICC**	**Paired-Samples** ***t*** **Test (t)**	***p*** **Value**
AL (mm)	SW600	83	83	24.140 ± 1.330	20.570–28.320	−0.020 ± 0.044	−0.030 to −0.011	−0.107 to 0.066	0.999	1.000	−4.238	<0.01
	LS900	83		24.119 ± 1.366	20.440–28.410							
R1 (mm)	SW600	83	83	7.980 ± 0.282	7.287–8.612	−0.031 ± 0.031	−0.037 to −0.024	−0.092 to 0.031	0.992	0.994	−8.897	<0.01
	LS900	83		7.950 ± 0.280	7.240–8.570							
R2 (mm)	SW600	83	83	7.633 ± 0.319	6.941–8.330	0.020 ± 0.045	0.010 to 0.030	−0.068 to 0.108	0.989	0.990	4.071	<0.01
	LS900	83		7.653 ± 0.321	6.900–8.330							
CCT (µm)	SW600	83	83	549.446 ± 32.956	491.000–637.000	4.386 ± 7.399	2.794 to 5.977	−10.117 to 18.888	0.996	0.978	5.400	<0.01
	LS900	83		553.831 ± 34.966	494.000–667.000							
AD (mm)	SW600	83	76	3.113 ± 0.261	2.280–3.570	0.088 ± 0.118	0.061 to 0.114	−0.143 to 0.319	0.865	0.899	6.538	<0.01
	LS900	76		3.201 ± 0.263	2.230–3.830							
LT (mm)	SW600	83	60	3.517 ± 0.238	2.970–3.940	−0.146 ± 0.148	−0.183 to −0.108	−0.436 to 0.145	0.716	0.785	−7.626	<0.01
	LS900	60		3.372 ± 0.171	2.880–3.690							
WTW (mm)	SW600	80	63	11.929 ± 0.440	10.690–12.820	0.140 ± 0.592	−0.009 to 0.289	−1.021 to 1.301	0.644	0.371	1.876	<0.05
	LS900	66		12.077 ± 0.600	8.660–13.690							
AL/CR	SW600	83	83	3.095 ± 0.147	2.800–3.540	−0.003 ± 0.012	−0.006 to −0.001	−0.026 to 0.020	0.995	0.997	−2.397	<0.05
	LS900	83		3.092 ± 0.149	2.796–3.546							

SW600, Swan SW600; LS900, Lenstar LS900; AL, Axial Length; R1, Radius in the flattest meridian; R2, Radius in the steepest meridian; CCT, Central Corneal Thickness; AD, Anterior Chamber Depth; LT, Lens Thickness; WTW, White-To-White distance; AL/CR, Axial Length/Corneal Radius. Difference in the means was computed by subtracting SW600 values from LS900 values; SD, Standard deviation; CI, Confidence interval; LoA, Limits of agreement; ρ, Spearman correlation coefficient; ICC, Intraclass Correlation Coefficient; *p* value, paired-samples *t* test. Valid N, the number of participants with valid measurements obtained by each device. Paired N, the number of participants with valid measurements from both devices for the corresponding parameter.

## Data Availability

The datasets generated and analysed in this study are not publicly available due to participant-privacy-related ethical restrictions. Anonymized study data are available from the corresponding author upon reasonable request.

## Abbreviations

AL	Axial length
CR	Corneal radius
R1	Corneal radius in the flattest meridian
R2	Corneal radius in the steepest meridian
AL/CR	Axial length-to-corneal radius ratio
CCT	Central corneal thickness
AD	Anterior chamber depth
LT	Lens thickness
WTW	White-to-white distance
OLCR	Optical low-coherence reflectometry
SLD	Superluminescent diode
SD	Standard deviation
CI	Confidence interval
ICC	Intraclass correlation coefficient
Sw	Within-subject standard deviation
CoR	Coefficient of repeatability
CoV	Coefficient of variation
LoA	Limits of agreement
